# Clinical outcomes after mix-and-match implantation of diffractive multifocal intraocular lenses with + 2.75 and + 4.00 diopter add powers

**DOI:** 10.1186/s12886-020-01460-7

**Published:** 2020-05-15

**Authors:** Jae Hyuck Lee, Hun Lee, Jin Ah. Lee, Aeri Yoo, Jae Yong Kim, Hungwon Tchah

**Affiliations:** Department of Ophthalmology, Asan Medical Center, University of Ulsan College of Medicine, 88, Olympic-Ro 43-Gil, Songpa-Gu, Seoul, 05505 South Korea

**Keywords:** Mix-and-match implantation, + 2.75 diopter add diffractive multifocal intraocular lens, + 4.00 diopter add diffractive multifocal intraocular lens

## Abstract

**Background:**

To evaluate the clinical outcomes of bilateral mix-and-match implantation of diffractive multifocal intraocular lenses (IOLs) with different add powers.

**Methods:**

We retrospectively reviewed the medical records of 18 patients who underwent bilateral mix-and-match implantation of diffractive multifocal IOLs with different add powers. Multifocal IOLs with add powers of + 2.75 diopters (D) and + 4.00 D were implanted into the patients’ dominant and nondominant eyes, respectively. At 1 and 3-month postoperatively, monocular and binocular visual acuity was measured using logMAR charts and manifest refraction was performed. Specifically, logMAR charts were used to measure uncorrected distance visual acuity (UDVA), uncorrected intermediate visual acuity (UIVA), uncorrected near visual acuity (UNVA), and corrected distance visual acuity (CDVA). Defocus curves, contrast sensitivity, and patient satisfaction were assessed at 3-month postoperatively.

**Results:**

Binocular logMAR measurements (mean ± standard deviation) at 3-month postoperatively were 0.01 ± 0.04 (UDVA), 0.16 ± 0.05 (UIVA), and 0.11 ± 0.07 (UNVA). Postoperative spherical equivalent was − 0.43 ± 0.35 D and − 0.39 ± 0.21 D in the dominant and nondominant eyes, respectively. Defocus curves showed significant differences between − 1.50 and − 4.00 D among binocular, dominant, and nondominant eye measurements, except between − 2.50 and − 3.00 D. Eyes implanted with + 2.75 and + 4.00 D IOLs showed good contrast sensitivity under photopic and mesopic conditions. Over 80% of patients reported high satisfaction with their near vision.

**Conclusions:**

Bilateral mix-and-match implantation of diffractive multifocal IOLs with add powers of + 2.75 D and + 4.00 D showed good near, intermediate, and far vision.

## Background

Technological developments, including the development of multifocal intraocular lenses (IOLs), have resulted in maximization of vision quality via cataract surgery. Bifocal IOLs show improvements in near and far distance visual acuity and emphasize near visual acuity [1]. In general, bifocal IOLs of the same type and with the same add power for each eye are bilaterally implanted [2].

However, several studies reported insufficient intermediate visual acuity with bilateral bifocal IOLs [3, 4]. The need for better intermediate distance visual acuity has led to efforts to improve the uncorrected intermediate visual acuity (UIVA) of these IOLs. For example, multifocal IOLs with extended depth of focus (EDOF) and trifocal IOLs have been developed and widely commercialized [5, 6]. However, trifocal IOLs showed more prominent background shadows than did bifocal IOLs, and EDOF IOLs have are limited in their ability to improve uncorrected near visual acuity (UNVA) [7, 8]. Thus, there are still no IOLs available that improve vision across all ranges.

Since the CONCERTO study used monovision approach with multifocal IOL [9], to overcome the limitations of available IOLs, efforts have been made to expand the range of vision by implanting different types of multifocal IOLs in each eye of a subject [10]. Several studies have reported a good range of vision after bilateral mix-and-match implantation of multifocal IOLs [2, 11, 12]. However, studies that evaluate clinical outcomes after implantation of diffractive multifocal IOLs with different add powers are scarce. Therefore, we aimed to evaluate clinical outcomes after implantation of TECNIS® IOLs with near addition powers of + 2.75 diopters (D) in the dominant eye and + 4.00 D in the nondominant eye.

## Methods

We conducted this retrospective observational case series with the approval of the Institutional Review Board of the Asan Medical Center and the University of Ulsan College of Medicine (Seoul, South Korea). The study adhered to the tenets of the Declaration of Helsinki and followed good clinical practice guidelines. All patients provided written informed consent to allow their medical information to be included for analysis and publication.

This retrospective study included all patients who underwent cataract surgery with bilateral mix-and-match implantation of a TECNIS® + 2.75 D multifocal IOL (ZKB00, Johnson & Johnson Vision, Santa Ana, CA, USA) into the dominant eye and a TECNIS® + 4.00 D multifocal IOL (ZMB00, Johnson & Johnson Vision) into the nondominant eye by one surgeon (HT) at the Cataract and Refractive Surgery Clinic of Asan Medical Center from March 2015 to February 2016. Tecnis ZKB00 is a diffractive bifocal IOL which has an anterior aspheric and diffractive profile posterior surface [13]. It features 50/50 light distribution between the distance and near foci independent of the pupillary size [13]. Tecnis ZMB00 shares same characteristics with ZKB00, which has + 4 D near addition (approximately + 3.0 D at the spectacle plane) [14]. Patients who met the following inclusion criteria were included: (1) older than 18 years, (2) preexisting corneal astigmatism less than + 1.00 D, and (3) visual acuity greater than 0.1 logMAR as measured with a potential acuity meter. Patients were excluded if they had (1) optical opacities or pathology on slit-lamp examination, (2) previous corneal surgeries, (3) ocular trauma, (4) intraocular surgery, (5) severe dry eyes, (6) corneal disease, (7) ocular infection, or (8) collagen vascular disease or other autoimmune diseases. Two subjects were excluded from the review because they had refractive surgery before. And for more precise verification of the surgery outcomes, within the limited data available, we additionally compared preoperative characteristics and postoperative visual acuities with bilateral TECNIS® 1-piece monofocal IOL (ZCB00, Johnson & Johnson Vision, Santa Ana, CA, USA) implantation group.

### Measurements

All subjects underwent comprehensive ophthalmological examinations preoperatively, including logMAR visual acuity measurements of monocular and binocular uncorrected distance visual acuity (UDVA), UIVA, UNVA, corrected distance visual acuity (CDVA), corrected intermediate visual acuity (CIVA), and corrected near visual acuity (CNVA). Preoperative assessments also included autorefraction and keratometry (Canon R-50, Canon USA Inc., Huntington, NY, USA), slit-lamp examinations (Haag-Streit, Gartenstadtstrasse, Köniz, Switzerland), biometry (IOL Master 500, Carl Zeiss Meditec, Jena, Germany), and corneal topography (Orbscan, Bausch & Lomb, Rochester, NY, USA). Each patient’s dominant eye was determined prior to surgery using the hole-in-the-card test wherein the patient looks at a target through a 1 in. hole in the center of a card held at one arm’s length, with only one eye open at a time, to determine which eye saw the target.

The ophthalmic examinations conducted at 1 and 3-month after surgery included logMAR measurements of monocular and binocular UDVA, UIVA, UNVA, and CDVA. Autorefraction and keratometry were also performed. Intermediate visual acuity was measured at 60 cm. Near visual acuity was measured at 33, 40, and 50 cm, with near visual acuity expressed as the average of visual acuity at these distances. In many previous studies, near VA was measured only at 40 cm [15, 16]. But we focused on that there were delicately different needs for near target distance in various situations such as reading books or watching mobile phones, therefore, we defined near VA more broadly as the average VA at 33, 40, and 50 cm in this study. In addition, monocular and binocular defocus curves were obtained at 3-month postoperatively by measuring monocular or binocular visual acuity at 4 m starting from distance correction and then defocusing with added lenses in half-diopter steps from − 4.50 D to 0.00 D. In monofocal IOL group, defocus curves were obtatined only binocularly according to our clinic’s own protocol .

Contrast sensitivity was measured monocularly under uncorrected condition at 3-month postoperatively, using the Functional Acuity Contrast Test of the Ophtec 6500 view-in test system (Stereo Optical Co, Inc., Chicago, IL, USA) with stimulus spatial frequencies between 1.5 and 18 cycles per degree under photopic (target luminance = 85 cd per square meter [cd/m^2^]) and mesopic (target luminance = 3 cd/m^2^) conditions.

Finally, patients were asked to complete a questionnaire regarding their overall satisfaction, the occurrence of visual symptoms, and their dependence on spectacles for near and far vision. Overall satisfaction was assessed using a 5-point Likert scale: 1 = very dissatisfied, 2 = dissatisfied, 3 = neither satisfied nor dissatisfied, 4 = satisfied, and 5 = very satisfied. Visual symptoms (glare, halo, and visual disturbances at night or in the dark) were scored on a 5-point scale from 1 (absent symptoms) to 5 (severe symptoms). Patients were also asked if they would recommend bilateral mix-and-match implantation of multifocal IOLs to their friends or relatives, with allowed responses being yes or no.

### Surgical technique

After instillation of topical anesthesia (0.5% proparacaine hydrochloride), the phacoemulsification surgery was performed. A continuous curvilinear capsulorrhexis marker with a 6.0-mm diameter was used to reference the corneal plane. The main clear corneal incision was made using a 2.2-mm keratome, followed by capsulorrhexis using a capsulorrhexis needle. Phacoemulsification was performed using either the Infiniti® or Centurion® phacoemulsifier (Alcon Laboratories, Inc., Fort Worth, TX, USA). Using an injector, a + 2.75 D multifocal IOL was implanted into the capsular bag of the dominant eye, and a + 4.00 D multifocal IOL was implanted into the capsular bag of the nondominant eye. The dominant eye was first implanted with Tecnis + 2.75 D multifocal IOL (ZKB00), and 1 week after surgery in the dominant eye, Tecnis + 4.00 D multifocal IOL (ZMB00) was implanted in the non-dominant eye. The target postoperative refraction was emmetropia in both eyes. All patients were administered 0.5% gatifloxacin ophthalmic solution (Gatiflo®, HANDOK, Seoul, South Korea) and prednisolone eye drops (Pred-Forte®, Allergan, Dublin, Ireland) for 1-month postoperatively. All of the above surgical protocols were equally applied to the bilaterally monofocal IOLs implanted group.

### Statistical analysis

Results are expressed as the mean ± standard deviation with range. Differences between preoperative and postoperative data were assessed using the Wilcoxon signed-rank test. Values for the defocus curves for both eyes, the dominant eye, and the nondominant eye were analyzed by the Kruskal-Wallis test with the Bonferroni correction. All statistical analyses were performed using SPSS® version 21 software (IBM, SPSS Inc., Chicago, IL, USA). Differences were considered statistically significant for *P* values of less than 0.05.

## Results

The study included 18 patients (10 female and 8 male), of mean age 65.8 ± 5.7 years (range, 55–76 years). Preoperative subject and ocular characteristics are summarized in Table 1. Statistically significant difference was not found in all parameters between bilateral multifocal IOL group and bilateral monofocal IOL group Table 2 shows preoperative and postoperative spherical equivalent (SE) and monocular visual acuity. At 3-month postoperatively, monocular logMAR UDVA, logMAR UIVA and logMAR UNVA of the dominant eye were 0.04 ± 0.05 (range: 0–0.20), 0.16 ± 0.05 (range: 0–0.50) and 0.17 ± 0.10 (range: 0–0.40), respectively. And monocular logMAR UDVA, logMAR UIVA and logMAR UNVA of the nondominant eye were 0.04 ± 0.05 (range: 0–0.30), 0.30 ± 0.12 (range: 0–0.50) and 0.11 ± 0.07 (range: 0–0.20), respectively. Postoperative monocular SE, logMAR UDVA, logMAR UIVA, logMAR UNVA and logMAR CDVA were all significantly better than preoperative values. Also significant differences were found between preoperative and postoperative binocular visual acuity for logMAR UDVA, logMAR UIVA, and logMAR UNVA in bilateral multifocal IOL group and only for logMAR UDVA in bilateral monofocal IOL group. (Tables 3 and 4). At 3-month postoperatively, binocular logMAR UDVA, logMAR UIVA and logMAR UNVA of bilateral multifocal IOL group were 0.01 ± 0.04 (range: 0–0.10), 0.16 ± 0.05 (range: 0–0.20) and 0.11 ± 0.07 (range: 0–0.20), respectively.

**Table 1:** Demographic and clinical characteristics of multifocal IOL group and monofocal IOL group

Parameter	Multifocal IOL group	Monofocal IOL group	*P* value*
Number of eyes / patients	36 / 18	34 / 17	
Sex (male / female)	8 / 10	8 / 9	
Age (years)	65.8 ± 5.7 (range: 55–76)	67.0 ± 5.9 (range: 56–78)	0.797
Mean corneal astigmatism (D)	0.60 ± 0.25 (range: 0–1.00) (dominant eye), 0.60 ± 0.30 (range: 0–1.00) (non-dominant eye)	0.58 ± 0.23 (range: 0–1.00) (dominant eye), 0.61 ± 0.27 (range: 0–1.00) (non-dominant eye)	0.688 0.724
Mean spherical equivalent (D)	0.55 ± 1.99 (range: − 5.75–2.25) (dominant eye), 0.21 ± 2.05 (range: − 5.50–3.50) (non-dominant eye)	0.61 ± 1.78 (range: − 4.75–2.00) (dominant eye), 0.25 ± 1.85 (range: − 5.00–3.25) (non-dominant eye)	0.556 0.473
Mean axial length (mm)	23.46 ± 1.08 (range: 20.84–26.00) (dominant eye), 23.52 ± 1.09 (range: 20.91–26.13) (non-dominant eye)	23.38 ± 0.99 (range: 21.01–25.87) (dominant eye), 23.50 ± 1.03 (range: 20.99–26.02) (non-dominant eye)	0.522 0.610

Results reported as means ± standard deviations

*D* Diopters, *IOL* Intraocular lenses

*Statistically significant at *P* < 0.05

**Table 2:** Refractive results and monocular visual acuity in patients with mix-and-match implantation of diffractive multifocal intraocular lenses with different add power

		MRSE	LogMAR UDVA	LogMAR UIVA	LogMAR UNVA	LogMAR CDVA
Preoperative	Dominant eye	0.55 ± 1.99 (range: − 5.75–2.25)	0.36 ± 0.31 (range: 0–1.30)	0.64 ± 0.25 (range: 0.30–1.20)	0.58 ± 0.30 (range: 0–1.00)	0.10 ± 0.10 (range: 0–0.30)
	Non-dominant eye	0.21 ± 2.05 (range: − 5.50–3.50)	0.38 ± 0.35 (range: 0–1.30)	0.62 ± 0.27 (range: 0.30–1.30)	0.58 ± 0.30 (range: 0–0.82)	0.13 ± 0.16 (range: − 0.10–0.52)
1-month postoperative	Dominant eye	−0.43 ± 0.44 (range: − 1.00–0)	0.06 ± 0.07 (range: 0–0.20)	0.16 ± 0.10 (range:0–0.50)	0.19 ± 0.09 (range: 0–0.50)	0.03 ± 0.06 (range: 0–0.20)
	*P* value*	< 0.001	0.002	< 0.001	0.003	0.021
	Non-dominant eye	−0.35 ± 0.37 (range: − 0.75–0.5)	0.07 ± 0.12 (range: 0–0.30)	0.29 ± 0.08 (range: 0.10–0.60)	0.13 ± 0.09 (range: 0–0.50)	0.02 ± 0.04 (range: 0–0.10)
	*P* value*	< 0.001	0.001	0.001	0.001	0.015
3-month postoperative	Dominant eye	−0.43 ± 0.35 (range: − 0.88–0)	0.04 ± 0.05 (range: 0–0.20)	0.16 ± 0.05 (range: 0–0.50)	0.17 ± 0.10 (range: 0–0.40)	0.01 ± 0.03 (range: 0–0.10)
	*P* value*	< 0.001	0.002	0.001	0.005	0.005
	Non-dominant eye	−0.39 ± 0.21 (range: − 0.75–0.25)	0.04 ± 0.05 (range: 0–0.30)	0.30 ± 0.12 (range: 0–0.50)	0.11 ± 0.07 (range: 0–0.20)	0.01 ± 0.03 (range: 0–0.10)
	*P* value*	< 0.001	0.003	0.002	0.002	0.019

Results reported as means ± standard deviations

*MRSE* Manifest refraction spherical equivalent, *UDVA* Uncorrected distance visual acuity; *UIVA* Uncorrected intermediate visual acuity, *UNVA* Uncorrected near visual acuity, *CDVA* Corrected distance visual acuity

*Compared with preoperative values

**Table 3:** Binocular visual acuity in multifocal IOL group and monofocal IOL group at 1 month postoperatively

	Preoperative	*P* value^+^	1-month postoperative	*P* value*	*P* value^+^
LogMAR UDVA (multifocal IOL group)	0.31 ± 0.31 (range: 0.10–0.60)	0.824	0.02 ± 0.04 (range: 0–0.10)	0.007	0.779
LogMAR UDVA (monofocal IOL group)	0.34 ± 0.28 (range: 0.10–0.60)		0.03 ± 0.05 (range: 0–0.20)	0.010	
LogMAR UIVA (multifocal IOL group)	0.56 ± 0.23 (range: 0.30–0.80)	0.659	0.14 ± 0.07 (range: 0–0.20)	0.007	0.004
LogMAR UIVA (monofocal IOL group)	0.49 ± 0.28 (range: 0.20–0.80)		0.37 ± 0.21 (range: 0.20–0.70)	0.102	
LogMAR UNVA (multifocal IOL group)	0.48 ± 0.25 (range: 0.20–0.80)	0.701	0.10 ± 0.10 (range: 0–0.30)	0.017	0.001
LogMAR UNVA (monofocal IOL group)	0.43 ± 0.26 (range: 0.20–0.80)		0.36 ± 0.22 (range: 0.20–0.70)	0.338	
LogMAR CDVA (multifocal IOL group)	0.09 ± 0.13 (range: 0–0.30)	0.871	0.01 ± 0.03 (range: 0–0.10)	0.084	0.921
LogMAR CDVA (monofocal IOL group)	0.10 ± 0.10 (range: 0–0.20)		0.01 ± 0.02 (range: 0–0.10)	0.077	

Results reported as means ± standard deviations

*UDVA* Uncorrected distance visual acuity, *UIVA* Uncorrected intermediate visual acuity, *UNVA* Uncorrected near visual acuity, *CDVA* Corrected distance visual acuity, *IOL* Intraocular lenses

*Compared with preoperative values

^+^Compared between multifocal IOL group and monofocal IOL group

**Table 4:** Binocular visual acuity in multifocal IOL group and monofocal IOL group at 3 month postoperatively

	Preoperative	*P* value^+^	3-month postoperative	*P* value*	*P* value^+^
LogMAR UDVA (multifocal IOL group)	0.31 ± 0.31 (range: 0.10–0.60)	0.824	0.01 ± 0.04 (range: 0–0.10)	0.011	0.702
LogMAR UDVA (monofocal IOL group)	0.34 ± 0.28 (range: 0.10–0.60)		0.03 ± 0.04 (range: 0–0.20)	0.009	
LogMAR UIVA (multifocal IOL group)	0.56 ± 0.23 (range: 0.30–0.80)	0.659	0.16 ± 0.05 (range: 0–0.20)	0.011	0.006
LogMAR UIVA (monofocal IOL group)	0.49 ± 0.28 (range: 0.20–0.80)		0.35 ± 0.22 (range: 0.20–0.70)	0.120	
LogMAR UNVA (multifocal IOL group)	0.48 ± 0.25 (range: 0.20–0.80)	0.701	0.11 ± 0.07 (range: 0–0.30)	0.017	0.002
LogMAR UNVA (monofocal IOL group)	0.43 ± 0.26 (range: 0.20–0.80)		0.37 ± 0.21 (range: 0.20–0.70)	0.305	
LogMAR CDVA (multifocal IOL group)	0.09 ± 0.13 (range: 0–0.30)	0.871	0.01 ± 0.03 (range: 0–0.10)	0.084	0.888
LogMAR CDVA (monofocal IOL group)	0.10 ± 0.10 (range: 0–0.20)		0.01 ± 0.03 (range: 0–0.10)	0.086	

Results reported as means ± standard deviations

*UDVA* Uncorrected distance visual acuity, *UIVA* Uncorrected intermediate visual acuity, *UNVA* Uncorrected near visual acuity, *CDVA* Corrected distance visual acuity, *IOL* Intraocular lenses

*Compared with preoperative values

^+^Compared between multifocal IOL group and monofocal IOL group

**Fig. 1:**
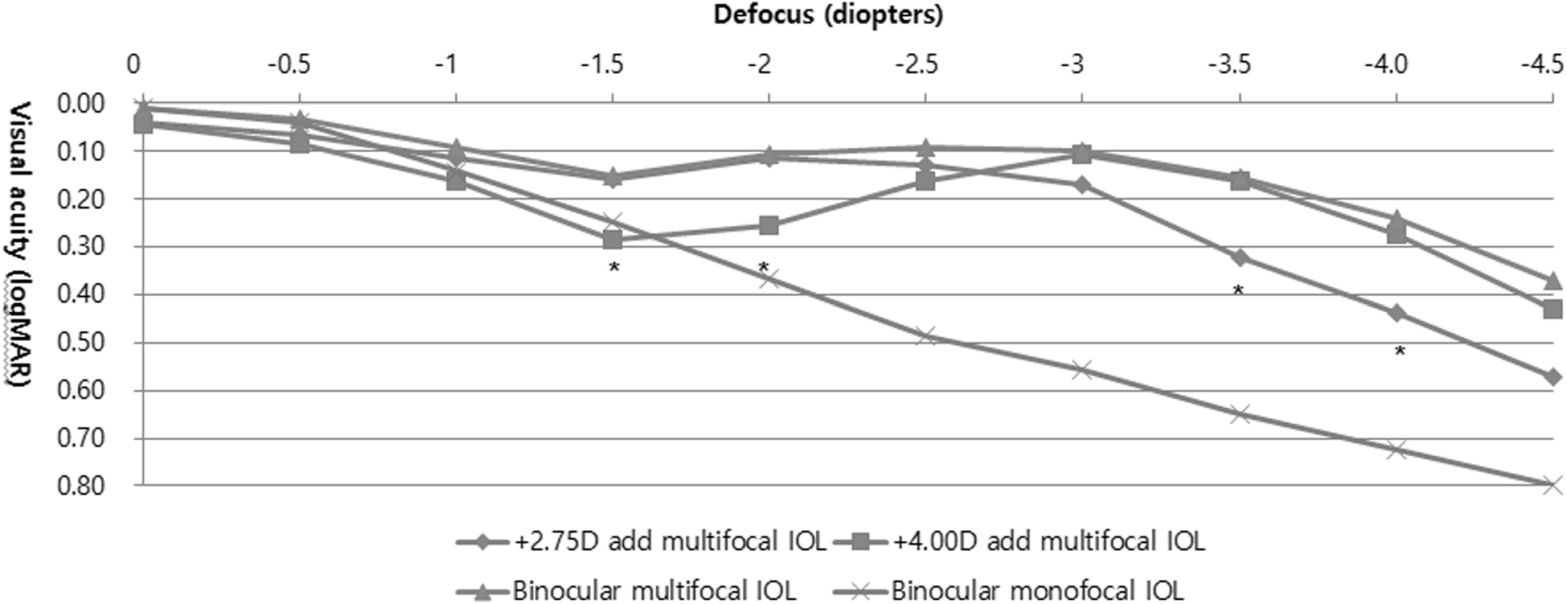
Binocular and monocular defocus curves for patients after bilateral mix-and-match implantation of diffractive multifocal intraocular lenses with +2.75 and + 4.00 diopter add powers, with binocular defocus curve of bilateral monofocal IOL implantations * *P* < 0.05. IOL, intraocular lens

The binocular and monocular defocus curves are shown in Fig. 1. Binocular defocus curves of bilateral multifocal IOL group showed a better range of postoperative vision when compared with each monocular defocus curve across all distances. Visual acuity between − 1.50 D and − 4.00 D differed significantly among the 3 defocus curves (+ 2.75 D multifocal IOL, + 4.00 D multifocal IOL, and binocular multifocal IOL), with the exception of between − 2.50 D and − 3.00 D (*P* = 0.001 for − 1.50 D, 0.003 for − 2.00 D, 0.003 for − 3.50 D, and 0.002 for − 4.00 D). Defocus curves between − 3.50 D and − 4.00 D differed significantly for comparisons of binocular multifocal IOL and + 2.75 D multifocal IOL monocular vision (*P* = 0.003 for − 3.50 D and 0.001 for − 4.00 D) and between − 1.50 D and − 2.00 D for comparisons of binocular multifocal IOL and + 4.00 D multifocal IOL monocular vision (*P* = 0.001 for − 1.50 D and 0.002 for − 2.00 D). Defocus curves between − 1.50 D and − 4.00 D also differed significantly for comparisons of + 2.75 D multifocal IOL and + 4.00 D multifocal IOL monocular vision, with the exception of between − 2.50 D and − 3.00 D (*P* = 0.001 for − 1.50 D, 0.007 for − 2.00 D, 0.006 for − 3.50 D, and 0.007 for − 4.00 D). Binocular defocus curves of bilateral monofocal IOL groups exhibited a visual acuity of 0.10 logMAR or better between 0 and − 0.5 D, then there was a sharp decrease in visual acuity over − 1.0 D.

**Fig. 2:**
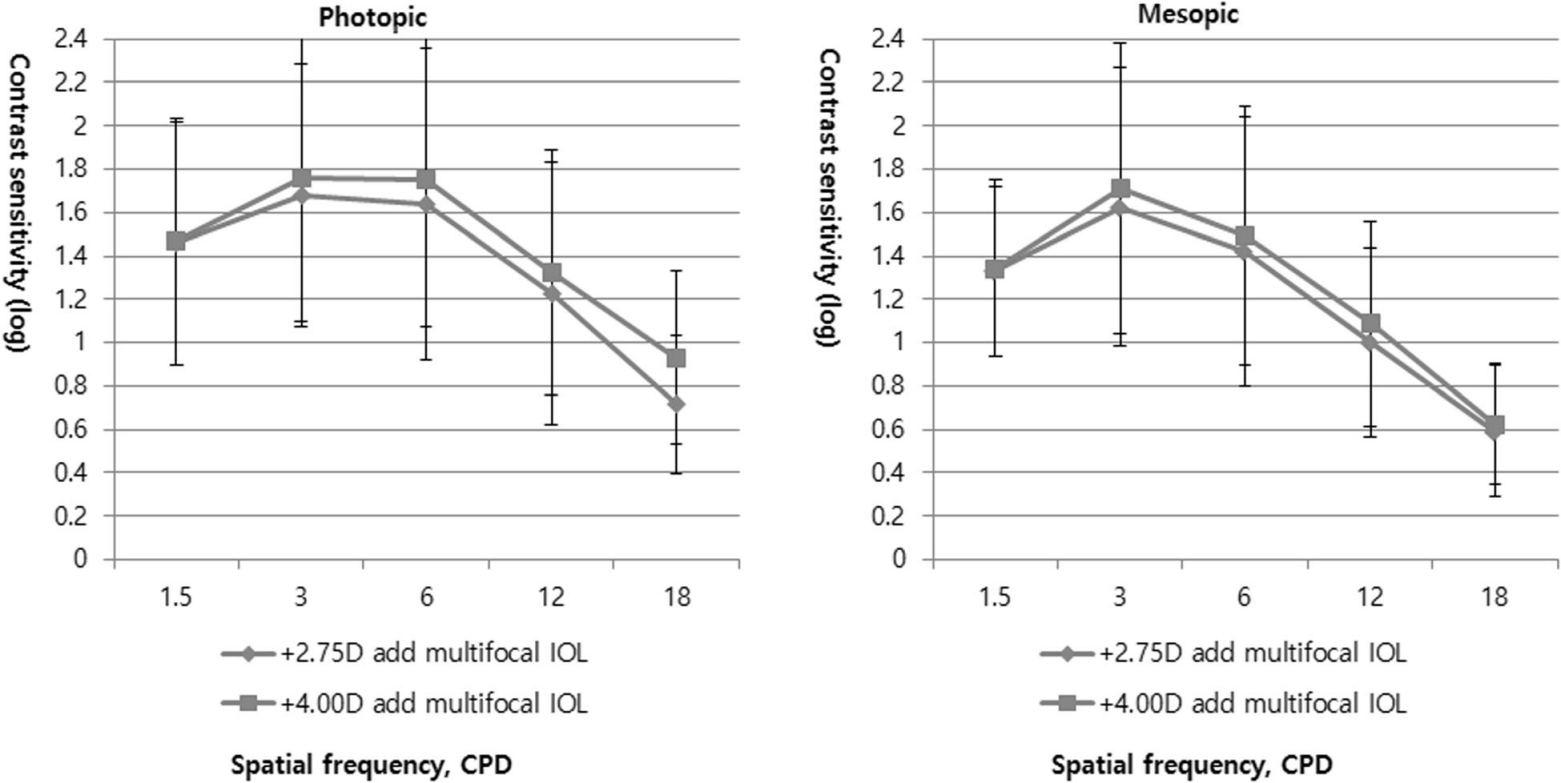
Contrast sensitivity test under photopic and mesopic conditions in patients with mix-and-match implantation of diffractive multifocal intraocular lenses with +2.75 and + 4.00 diopter add powers. CPD, cycles per degree

As shown in Fig. 2, contrast sensitivity was demonstrated under both photopic and mesopic conditions in eyes implanted with + 2.75 D and + 4.00 D multifocal IOLs, with no significant differences at any spatial frequency.

Sixteen subjects completed satisfaction questionnaires at 3-month postoperatively. Thirteen subjects (81.3%) reported that they were satisfied or very satisfied with their near vision, with an average satisfaction score of 4.4 ± 0.9 (range: 2–5). Only one subject (6.3%) reported occasionally needing glasses for near vision after surgery. Regarding the rate of visual symptoms, 4 (25.0%) subjects reported glare and halo symptom scores > 3 (average score: 2.4 ± 0.9) (range: 1–5) and 3 (18.8%) patients scored symptoms of visual disturbances at night or in the dark at ≥3 (average score: 2.3 ± 0.9) (range: 1–5) (Table 5).

**Table 5:** Results for questionnaire about overall satisfaction, visual symptoms and dependence on spectacles

Questionnaire	Response (average score/rate)
Overall satisfaction	4.4 ± 0.9 (range: 2–5), very satisfied or satisfied: 81.3%
Needing for near glasses after surgery	4.8 ± 0.6 (range: 3–5), occasionally need near glasses: 6.3%
Glare and halo symptoms	2.4 ± 0.9 (range: 1–5), over score 3: 25.0%
Visual disturbance at night or dark place	2.3 ± 0.9 (range: 1–5), over score 3: 18.8%
Recommendation for mix-and-match implantation	Yes: 93.8%

Results reported as means ± standard deviations

*IOL* Intraocular lenses

Satisfaction scale; 5 = very satisfied; 1 = very dissatisfied; Need for near glasses; 5 = not at all; 1 = always needed. Scale of discomfort due to visual symptom; 5 = severe symptoms; 1 = absent symptoms

## Discussion

In the current study, we demonstrated that bilateral mix-and-match implantation of multifocal IOLs with add powers of + 2.75 D and + 4.00 D showed good near, intermediate, and far vision. We implanted multifocal IOL with add power of + 4.00 D into the nondominant eye for near visual acuity, which was relatively high add diopter compared to previous studies, based on the fact that Asians have lower amplitudes of accommodation and thus need higher add powers compared to Caucasians [17]. Previously, patients who underwent implantation of bilateral + 4.00 D multifocal IOLs have reported problems with intermediate vision, although their near visual acuity was good [16]. Bilateral implantation of + 2.50 D and + 3.00 D multifocal IOLs resulted in good near vision and noninferior intermediate and distance vision compared with bilateral implantation of + 2.50 D multifocal IOLs [2]. Unilateral implantation of TECNIS® + 2.75 D, + 3.25 D and + 4.00 D multifocal IOLs resulted in similar monocular UDVA, and UNVA was best in patients who underwent diffractive multifocal IOL implantation with add power of + 2.75 D at 50 cm [18]. Bilateral implantation of the TECNIS® + 2.75 D and + 3.25 D multifocal IOLs resulted in good binocular UIVA (0.07 ± 0.11 logMAR) but relatively inferior binocular UNVA (0.25 ± 0.11 logMAR) [11]. On the basis that mix-and-match implantation of multifocal IOLs with different add powers may be more beneficial than bilateral implantation of multifocal IOLs with the same add power for subjects who desire spectacle independence, we evaluated clinical outcomes after bilateral mix-and-match implantation of diffractive multifocal IOLs with different add powers. A + 2.75 D multifocal IOL was implanted into the dominant eye, and a + 4.00 D multifocal IOL was implanted into the nondominant eye.

The spectacle plane add power of multifocal IOLs differs from the IOL plane add power. For example, the spectacle plane add powers of the TECNIS® + 2.75 D, + 3.25 D, and + 4.00 D multifocal IOLs are + 2.01 D, + 2.37 D, and + 3.00 D, respectively. In the present study, depending on the spectacle plane add powers of the IOLs, visual acuity in the 0.00 D to − 3.00 D range of binocular defocus curves was 0.1 logMAR or better. Binocular visual acuity at − 3.50 D was better than 0.2 logMAR. On the other hand, a previous study found that the second peak of the binocular defocus curve was at − 2.00 D, and the visual acuity at − 2.50 D was about 0.0 logMAR [11]. Then, visual acuity dropped sharply at values below − 2.50 D, being 0.2 logMAR and 0.3 logMAR at − 3.00 D and − 3.50 D [11].

Recently introduced TECNIS® EDOF IOLs provide an elongated focal area but not multiple foci. Therefore, these IOLs could provide better intermediate vision than other currently available multifocal IOLs. However, UIVA from our study was better than that of subjects who underwent bilateral implantation of TECNIS® EDOF and TECNIS® + 4.00 D multifocal IOLs [19]. Although binocular defocus curves showed that the visual acuity in the 0.00 D to − 1.50 D range in a previous study were similar to those in the present study, the earlier study found that visual acuity decreased at values below − 2.00 D [20]. Several studies have evaluated methods to overcome the inferior UNVA following implantation of EDOF IOLs [9, 20, 21]. For example, according to the CONCERTO prospective case series study in which EDOF IOLs were implanted into both eyes of 411 subjects, 299 had emmetropia target (non-monovision) and 112 had micro-monovision. The mean UNVA of the micro-monovision and non-monovision groups were 0.17 logMAR and 0.21 logMAR, respectively, with the former being inferior to UNVA in our study [9]. To sum up, bilateral mix-and-match implantation of diffractive multifocal IOLs with add powers of + 2.75 D and + 4.00 D can be the good alternatives of implantion of EDOF IOL for intermediate visual acuity, with better near visual acuity, too.

According to the results for contrast sensitivity test of the current study, eyes implanted with diffractive multifocal IOLs with + 4.00 D add powers showed better contrast sensitivity than eyes implanted with diffractive multifocal IOLs with + 2.75 D add powers, albeit the differences were not significant. Contrast sensitivity under photopic and mesopic conditions without glare was similar to the results of previous studies of bilateral implantation of the TECNIS® bifocal IOLs and of bilateral implantation of an EDOF IOL and a TECNIS® + 4.00 D multifocal IOL [2, 18]. Furthermore, similar results at all spatial frequencies under photopic conditions were reported between the mix-and-match group and the bilaterally + 2.50 D implanted group [10].

In terms of subject satisfaction, we found that 25.0% had moderate glare and halo symptoms, while 18.8% reported night vision problems. These findings were inferior to those of the CONCERTO study [8]. On the other hand, these results are superior to those of previous studies with implantation of bilateral multifocal IOLs [22, 23]. In addition, the rate of spectacle independence was higher in our study than in the CONCERTO study. Our findings that 93.8% of subjects reported that they would recommend mix-and-match implantation of diffractive multifocal IOLs with + 2.75 D and + 4.00 D add powers to their friends and relatives indicated that despite some visual problems, these symptoms did not have a significant impact on overall satisfaction.

Our study had several limitations, including the small number of eyes and the lack of a control group. Nonetheless, we demonstrated that UIVA and UNVA following mix-and-match implantation of diffractive multifocal IOLs with + 2.75 D and + 4.00 D add powers was not inferior to those following bilateral implantation of EDOF IOLs or bilateral implantation of an EDOF IOL and a diffractive trifocal IOL [24, 25]. Further research is needed on the bilateral implantation of other types of multifocal IOLs, especially trifocal and EDOF IOLs, to determine which combination of IOLs could provide superior UIVA, extended visual acuity range on defocus curves, and high spectacle independence at all distances.

## Conclusions

In summary, we evaluated clinical outcomes after mix-and-match implantation of diffractive multifocal IOLs with + 2.75 D and + 4.00 D add powers and demonstrated good near, intermediate, and far vision with a high degree of patient satisfaction. Therefore, mix-and-match implantation of + 2.75 D and + 4.00 D multifocal IOLs can be a good option for subjects who do not want to depend on glasses after cataract surgery.

## Data Availability

The datasets of the current study are available from the corresponding author on reasonable request.

## Abbreviations

CDVA: Corrected distance visual acuity
CPD: Cycles per degree
D: Diopters
EDOF: Extended depth of focus
IOL: Intraocular lenses
logMAR: Logarithm of the minimal angle of resolution
MR: Manifest refraction
SE: Spherical equivalent
UDVA: Uncorrected distance visual acuity
UIVA: Uncorrected intermediate visual acuity
UNVA: Uncorrected near visual acuity
